# Acoustophoretic assembly of millimeter-scale Janus fibers†

**DOI:** 10.1039/c9ra09796a

**Published:** 2020-01-02

**Authors:** Meghana Akella, Soheila Shabaniverki, Jaime J. Juárez

**Affiliations:** Department of Mechanical Engineering, Iowa State University Ames Iowa 50011 USA jjuarez@iastate.edu; Center for Multiphase Flow Research and Education, Iowa State University 2519 Union Drive Ames IA 50011 USA

## Abstract

This article presents a method for the assembly of millimeter-scale Janus fibers using acoustophoresis as an assembly mechanism. An acoustic flow cell mounted to a 3D printer combines acoustophoresis and additive manufacturing in a unique approach that allows for the assembly of textured Janus fibers. A dispersion consisting of polymethylmethacrylate (PMMA) filler particles in a UV curable polymer resin is passed through an acoustically excited capillary tube. To fundamentally understand this process, we develop a suspension balance model that accounts for acoustophoresis and concentration-driven shear-induced diffusion. Once assembled, the particle-polymer dispersion is cured using UV illumination to create a polymer composite fiber with particles immobilized on one side in a Janus-like configuration. The Janus fiber is observed to modify the light transmission profile when rotated on an optical microscope stage. Tensile measurements of the fiber show that the Young's modulus of the Janus fiber (50.5 MPa) is approximately twice that of a fiber fabricated from the polymer alone (24.7 MPa). The process we describe here could serve as a pathway for the fabrication of a variety of functional Janus fibers with possible applications to wearable textiles, soft robotics or surgical sutures.

## Abstract 2

This article presents a method for the assembly of millimeter-scale Janus fibers using acoustophoresis as an assembly mechanism.

## Graphical abstract

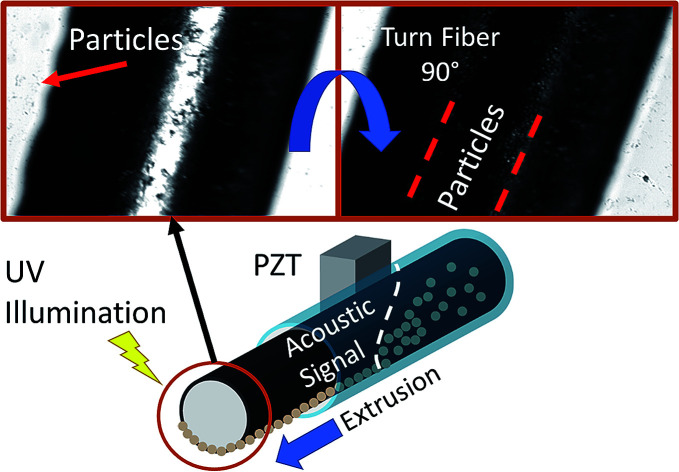

## 1. Introduction

Structurally anisotropic materials are used to encode specific chemical^1^ or mechanical^2^ properties that form the basis for a variety of applications. One class of anisotropic structures that have recently received attention as a functional material are known as Janus fibers.^3^ These Janus fibers have been incorporated into textiles to serve as water repellant coatings,^4^ electrically conducting structures for wearable electronics,^5^ and thermally conducting membranes for personalized thermal management.^6^ Membranes fabricated with Janus fibers can be used in traditional chemical engineering operations such as distillation^7^ or separation.^8^ Janus fibers may also serve as substrates for catalytic reactions.^9^

While there are many potential applications for Janus fibers, the processes for fabricating these types of materials remain limited. Electrohydrodynamic processes, such as electrospinning, are the most conventional method for fabricating Janus fibers. In this process, two different types of polymers are blended together in a nozzle and deposited to an electrically charged surface.^10^ An ancillary electrohydrodynamic process is to absorb particles to a single polymer phase during electrospinning.^11^ Janus fibers can also be formed by drop casting particles to a textile surface.^12^ Co-flowing microfluidic devices are also used to draw a two different polymer phases into a single strand, which is cured using UV illumination.^13,14^

One process that is not as prevalent in Janus fiber fabrication is the use of external fields (*e.g.*, magnetic, electric, acoustic) to drive the segregation of one phase from another. Where most of the processes described above rely on direct absorption of particles to form the Janus fiber, external fields act on the particle phase to assemble a Janus-type structure.^15^ External fields have the benefit directing particle assembly on a timescale that is faster than absorption by diffusion. In effect, external fields increase the Peclet number of the system, indicating that the rate of particle advection is dominant during the assembly process in comparison to Brownian motion.^16^ Process control of particle assembly in the polymer medium is possible by tuning field frequency or intensity to create a wide variety of microstructures,^17,18^ opening up the possibility for the development of new types of Janus materials with engineered microstructures.

This article seeks to address this apparent gap by using acoustics and sedimentation as part of a dual external field process for assembling Janus fibers. Our method relies on an acoustofluidic device assembled from off-the-shelf capillary tubes and piezoelectric elements, which simplifies the Janus fiber assembly process. The acoustofluidic device is mounted to a 3D printer based on a CNC gantry platform, where it acts as a type of 3D print head for the fabrication of Janus fibers. The operation of this 3D print head is fundamentally examined by using suspension balance modeling to account for sphere packing due to acoustophoresis and shear-induced diffusion due to hydrodynamic collisions between particles. The model is used to demonstrate how suspension balance modeling can be used as a method for measuring pressure in acoustophoretic systems. The Janus fibers formed by this process are observed to block the optical transmission of light when rotated on a microscope stage, a property that could find potential use in optical encoding and identification. Tensile testing of the Janus fibers exhibit a nearly two-fold increase in Young's modulus when compared fibers consisting of bare polymer.

This work builds upon previous applications of acoustic field assembly to additive manufacturing of composite materials^19–22^ by introducing this approach to the fabrication of Janus fibers. The suspension balance model used in this article represents the first time an equation of state is applied to describe the competition between acoustophoretic assembly of spheres and shear-induced diffusion. Our results also clearly demonstrate that the microstructure of filler particles influences the mechanical properties of the Janus fiber, which serves as a possible step towards tailoring composites for specific functional material applications.^23^ The method described in this article could form a framework for controlling filler particle microstructure for applications that require tunable properties such as fibers that undergo shape transformation due to local heating induced by catalytic reactions,^24^ anisotropic bending moments in a magnetic field that are the result of particle alignment within the fiber,^25^ or specific decay profiles for the controlled release of a drug.^26^

## 2. Materials and methods

### 2.1. Acoustic cell fabrication and operation

**Fig. 1:**
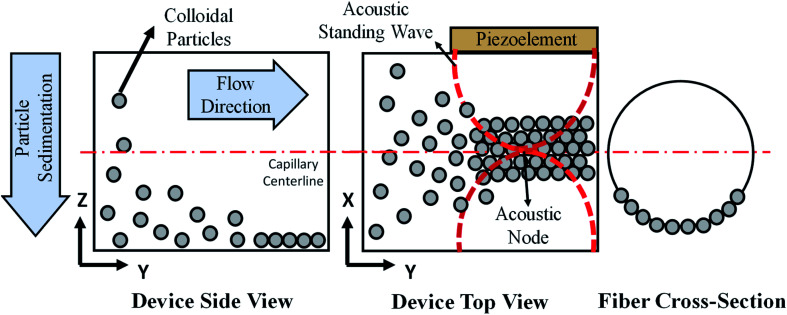
Schematic of the acoustic flow cell and acoustic assembly process. A combination of sedimentation and acoustic field assembly form a Janus fiber structure with particles assembled on only one side of the fiber.

The print head we used for continuous assembly of Janus fibers was based on our previous work with acoustophoretic assembly.^27^Fig. 1 is a schematic diagram showing the operation of the print head used to assemble Janus fibers. A complete description of the device can be found in our previous publication. Briefly, we used a square glass capillary (Vitrocom, 8100) with an inner side length of 1 mm and a 0.2 mm wall thickness that we affixed to two PDMS (Ellsworth Adhesives, Part# 184 SIL ELAST KIT 0.5 KG) pillars with a 5 mm diameter and 4 mm thickness using 5 minute epoxy (Devcon). The piezoelectric element was a lead zirconate titanate type (APC International Inc., P-30.00 mm-5.00 mm-1.00 mm-841 WFB), which was attached to the capillary using a high strength epoxy (JB Weld Epoxy Steel Resin) and cured for 24 hours. Silicone tubing (VWR International, catalog# 16211-316) was attached to either end of the capillary to introduce and extrude the Janus fiber.

The piezoelement was driven using a sine wave output from a function generator (Agilent, 33220A). An RF amplifier (Electronics & Innovation, 210L), connected in series with the function generator, was used to amplify the sine wave input into the piezoelement. The amplitude and frequency of the signal from the amplifier was monitored using an oscilloscope (Tektronix, TBS 1052B-EDU). A signal attenuator (Tektronix P2220 Voltage Probe) was used on the oscilloscope to reduce the possibility of damage on the oscilloscope due to high voltage. Two dummy loads of 1 W (Tektronix 011-0049-01) and 50 W (Pasternack PE6234) were used with the function generator and the RF amplifier, respectively, to ensure that the circuit remained closed at all times. All voltages in this article are reported as peak-to-peak voltage (*i.e.*, the difference between the peak and trough of the input sinusoidal waveform).

### 2.2. Sample preparation

In order to measure the acoustic pressure and calibrate our suspension balance model, we use 15 μm polystyrene beads sourced from Polysciences (product number 18328-5). We added 100 microliters of stock polystyrene bead solution to 1 mL deionized water sourced from an ARIES High Purity Water System with a 0.2 micron filter (Aries Filterworks).

After using our model to find acoustic pressure in our system, we applied this measurement to show that the model can predict microstructure for particles assembled in a polymeric fluid. The ESI† contains additional details on several different types of filler particles examined by the authors. These particles demonstrate the flexibility of our acoustic print head to operate with different types of materials, although this article focuses on applying polymethylmethacrylate (PMMA) as a filler particle. We selected PMMA for its versatility to act as a filler material for a variety of applications, including dental resin,^28^ bone cement^29^ and dielectric composites for capacitors.^30^ The PMMA used in this article was sourced from Cospheric (product number PMPMS-1.2 27-32um) and had a nominal size range of 27 μm to 32 μm in diameter.

A UV-curable resin was purchased from Sigma Aldrich (CPS 1030 UV-A) to serve as the polymeric fluid. The resin has a nominal kinematic viscosity of 70 cSt, but was diluted using acetone and isopropyl alcohol in a 1 : 1 : 1 mass ratio. Based on the Refutas index for determining the viscosity of fluid mixtures,^31^ we estimate that this mixture has a dynamic viscosity of approximately 1.76 mPa s. The mixture is slightly more viscous than water, which improves our ability to pump this fluid through the acoustic flow cell. The addition of solvents to the resin does not inhibit curing. For each experiment, 1 gram of dry PMMA particles was added to 10 mL of resin mixture to create dispersions with a bulk volume fraction of 7.8% for our experiments.

### 2.3. Optical microscopy

Acoustic pressure measurements in water were performed on an inverted optical microscope (Olympus IX70) with 10× magnification. Videos were recorded using a scientific CMOS (sCMOS) camera (QImaging, Optimos). One thousand frames of video were taken at a frame rate of 10 frames per second. Each pixel in the recorded video was measured to be 885.6 nm with a stage micrometer. The videos were processed and analyzed using the MATLAB Image Processing Toolbox (Mathworks). Each frame was filtered to remove noise before identifying centroid locations. The centroid locations are written to a text file and saved for the density distribution analysis.

### 2.4. Janus fiber assembly

**Fig. 2:**
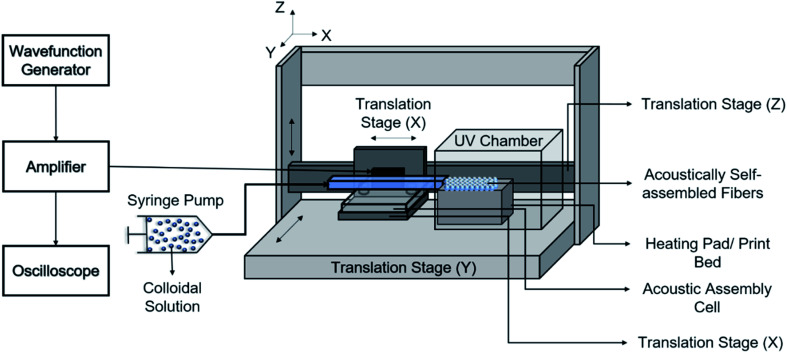
Schematic diagram of the extrusion process used to create the Janus fibers.

The acoustic print head (Fig. 1) is mounted to a CNC gantry (Zen Toolworks CNC Carving Machine DIY Kit 12x12 F8), which acts as a 3D printer for Janus fiber extrusion. A syringe pump (GenieTouch, Lucca Technologies) is used to introduce the PMMA particle-polymer dispersion to the acoustic print head as shown in Fig. 2. The PMMA particles are assembled using 85 V as a direct input from the RF amplifier. A fixed frequency of 848 kHz was identified as the operating resonance frequency for our acoustic print head. A single acoustic node forms at the center of the glass capillary, which drives PMMA particles to assemble at this location as a result of acoustophoresis. The Janus fiber experiments were conducted at a fixed flow rate of 2 mL h^−1^ using the syringe pump. The acoustic print head extrudes material directly into a UV illuminated chamber (SUNUV SUN2C 48 W LED UV lamp). The 3D printer moves with a feed rate of 1 mm s^−1^ in the *X* and *Y* directions to provide space for the Janus fiber to form. The acoustic print head is completely shielded from the UV chamber using craft paper to avoid internal curing.

## 3. Theory

The particle assembly in our print head is driven by acoustophoresis, where the particles migrate due to a non-uniform acoustic pressure gradient.^32^ The characteristic acoustophoretic force acting on the particles is,^33^

1 *F*_ac_ = 2*kU*_o_*Φ* sin(2*kx*)

where *k* = π/*w* is the wavenumber for a device with a single standing wave, *w* is the capillary width, *U*_o_ is the acoustic energy amplitude, *Φ* is the acoustic contrast factor that is determined from the relative different between particle and medium densities and compressibilities and *x* is the position of the particle relative to the centerline of the capillary. The acoustic energy amplitude is related to the applied pressure by,2
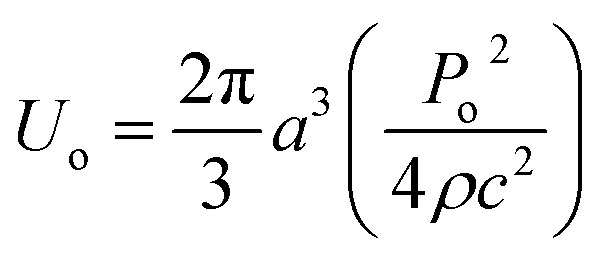
where *P*_o_ is the pressure amplitude of the acoustic field, *a* is the size of the particle confined by the acoustic field, *ρ* is the fluid density and *c* is the speed of sound in the fluid.

When a dispersion of particles is subjected to an acoustic field, the equilibrium material balance for the system yields,^34^

3 *J*_s_ + *J*_ac_ = 0

where *J*_s_ is the flux of material due to shear-induced stresses on the dispersion and *J*_ac_ is the acoustic flux. The acoustic flux is defined as, *J*_ac_ = *F*_ac_*ϕ*/6π*μ*_f_*a*, where *μ*_f_ is the fluid viscosity and *a* is the particle radius. Substituting eqn (1) into this expression leads to,

4 *J*_ac_ = *u*_ac_*ϕ* sin(2*kx*)

where *u*_ac_ = 2*kU*_o_*Φ*/6π*μ*_f_*a* represents the acoustophoretic velocity amplitude of a single particle.

The shear-induced flux is derived by defining an effective thermodynamic pressure that acts on the particle ensemble,^35^5
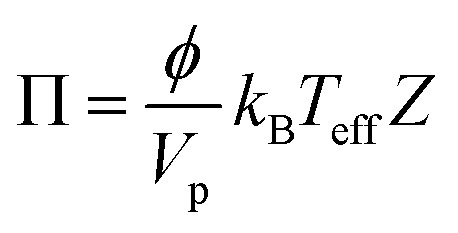
where *ϕ* is the area fraction of the particles that have sedimented to the bottom of our device, *V*_p_ is the average particle volume, *k*_B_*T*_eff_ = *μ*_f_*ΓV*_p_/9 is an effective thermal energy that arises from collisions between particles in shear flow, *Γ* = 6*Q*/*w*^3^ is the shear rate,^36^*Q* is fluid flow rate, and *Z* is the thermodynamic compressibility factor. The thermodynamic force resulting from the particle concentration gradient is,^37^
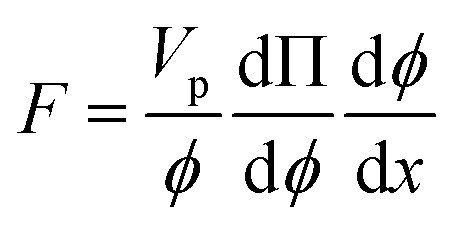


We can substitute eqn (5) into this expression to find the effective thermodynamic force acting on the particle ensemble due to shear in the presence of a concentration gradient,6
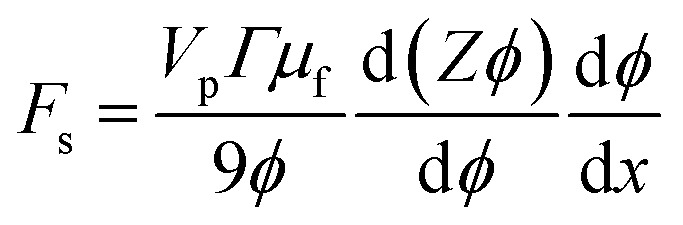


The shear-induced flux can be obtained in a manner similar to the acoustic flux, *J*_s_ = *F*_s_*ϕ*/6π*μ*_f_*a*. Substituting eqn (6) into this expression for flux allows us to arrive at a simplified expression for shear-induced flux, assuming the particle is spherical,7
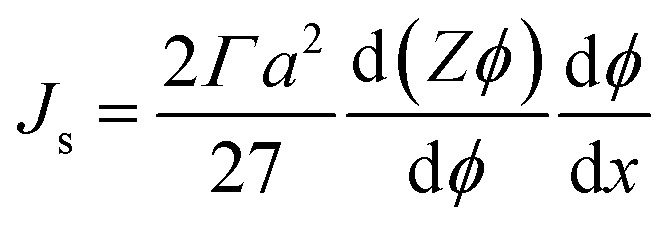


The equilibrium material balance can be expressed by substituting eqn (4) and (7) into eqn (3),
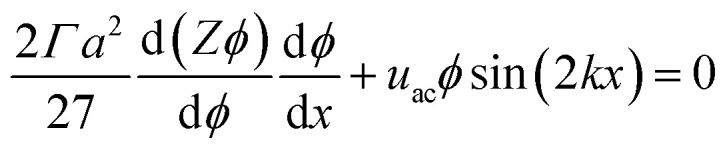


The equilibrium material balance can be simplified by introducing the non-dimensional parameters *x̂* = *x*/*w* and Pe = *u*_ac_*w*/*Γa*^2^. Substituting the dimensionless position and acoustic Peclet number into the equilibrium material balance leads to,8
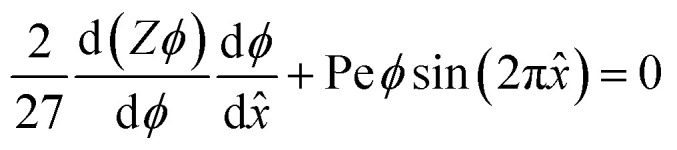


In our initial experiments in water, the assembled particles form a monolayer. Based on this observation, we assume that the compressibility factor for the monolayer is represented by a hard disk equation of state,^38^*Z* = (1 − *ϕ*)^−2^. Closing eqn (8) with an equation of state allows us to implement a forward Euler numerical scheme^17^ to find area fraction as a function of position. The parameters used to numerically integrate eqn (8) are shown in Table 1.

**Table 1:** Parameters used to evaluate the density distribution (eqn (8)) for particles confined at the acoustic node for both water and polymeric fluid experiments

Parameter	Description	Values
*w* (μm)	Capillary width	1000
*Φ*	Acoustic contrast factor, polystyrene	0.23
	Acoustic contrast factor, PMMA	0.34
2*a* (μm)	Particle diameter, polystyrene	15
	Particle diameter, PMMA	29.5
*ρ* (kg m^−3^)	Water density	1000
	Polymeric fluid density	917
*c* (m s^−1^)	Speed of sound in water	1480
	Speed of sound in polymeric fluid	1512
*μ*_f_ (mPa s)	Water viscosity	0.89
	Polymeric fluid viscosity	1.77
*Q* (mL h^−1^)	Flow rate for water	1
	Flow rate for polymeric fluid	2

## 4. Results and discussion

**Fig. 3:**
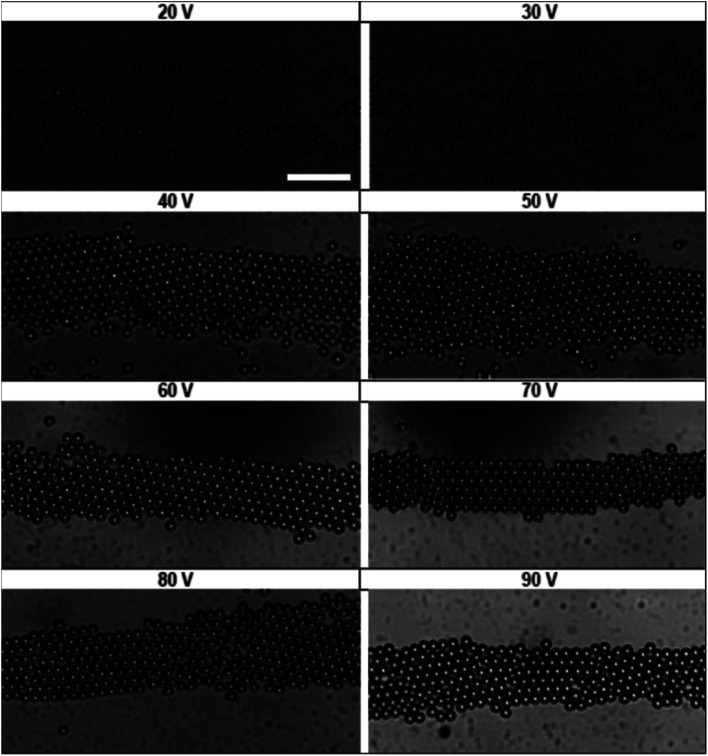
State diagram showing the various stages of acoustic assembly subject to different voltage inputs. Scale bar is 100 μm.

In order to measure the acoustic pressure in our device and calibrate our suspension balance model, an aqueous dispersion of 15 μm polystyrene particles were introduced to the acoustic print head at a flow rate of 1 mL h^−1^. We generated an acoustic field of fixed frequency (830 kHz) and varied the peak-to-peak voltage input from 20 V to 90 V in 10 V increments to change the acoustic pressure. The distributions that resulted from this pressure were observed experimentally in Fig. 3. We observed that applied voltages below 40 V do not exhibit particle assembly. Once the voltage reaches 40 V and 50 V, the particles transition to a randomly close-packed polycrystal with multiple domains present. The increased acoustic pressure at 60 V and 70 V lead to crystalline microstructures with few defects. At 80 V and 90 V, we observe that the particles transition back to a randomly close-packed microstructure. At voltages approaching 90 V, we observe that the particles begin to slow down, indicating that particles are in the process of becoming trapped at the acoustic node.

**Fig. 4:**
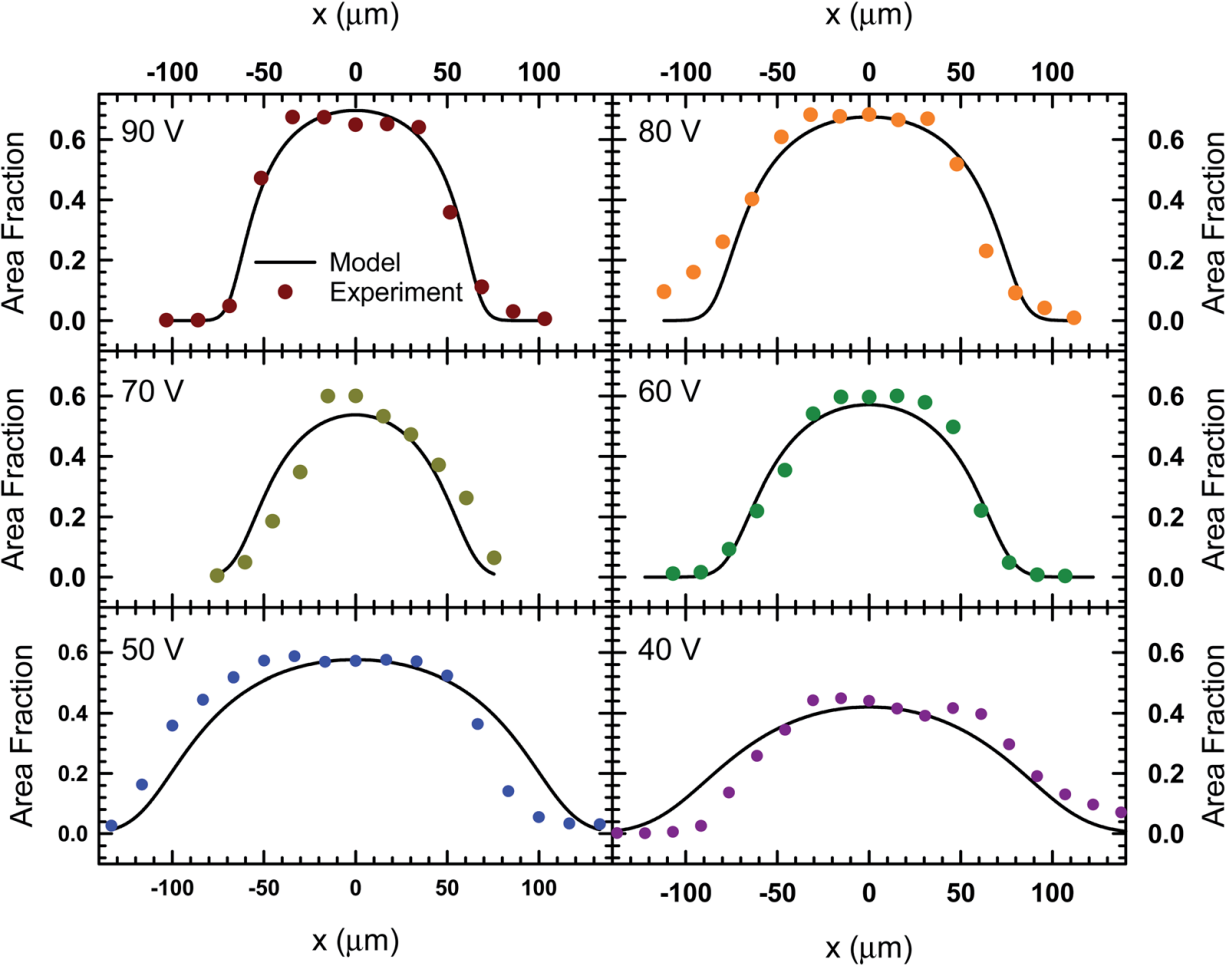
The local area fraction as a function of lateral position, *x*, in the acoustofluidic device subject to various applied voltages. The circles represent experimental data and the lines represent the fit obtained from the model described by eqn (8).

The images in Fig. 3 represent a single image from a 1000 frame video captured over a 100 second period. The videos recorded for these experiments were analyzed using the MATLAB image processing toolbox to identify unique particle centers. The tracking data is used to construct a histogram of cross-channel particle positions (*i.e.*, *x* values) for all of the data sets between 30 V and 90 V. We found that the 20 V data set exhibit significant variation in area fraction due to fluctuations in particle distribution. The local area fraction is calculated using the expression, *ϕ*_i_ = π*a*^2^*N*_i_/*N*_f_*w*_b_*L*_f_, where *N*_i_ is the number of particles observed in bin i of the histogram constructed for the total duration of the video, *N*_f_ is the number of frames in the video, *w*_b_ is the bin width and *L*_f_ is the physical length of the frame in the *y* direction. For our analysis, we set the bin width equal to the diameter of the particles used in Fig. 3 (*w*_b_ = 15 μm), while the frame length for our experiments was 1100 μm. This calculation allowed us to express our histograms as local area fraction as a function of position, shown as solid symbols in Fig. 4.

**Fig. 5:**
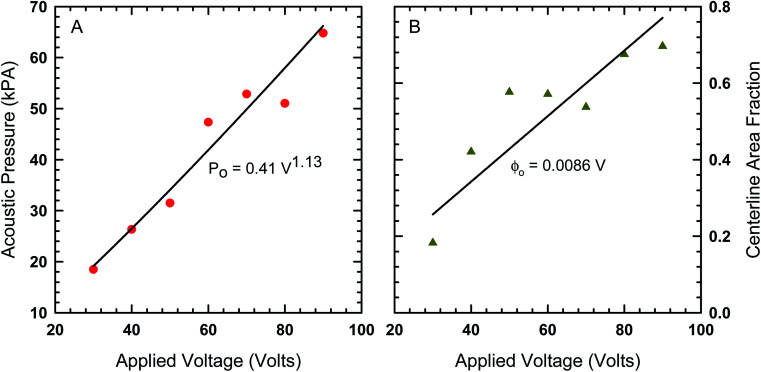
(A) The acoustic pressure and (B) centerline area fraction as a function of voltage determined by fitting the model in eqn (8) to the area fraction data in Fig. 4.

In order to find the best fit to data using our model from eqn (8), the acoustic Peclet number and the centerline area fraction at the acoustic node (*x* = 0) were used as adjustable parameters. The results from this process are shown in Fig. 4 as solids lines, which show that, despite some fluctuations in local area fraction, the model does match with experiment very closely. The acoustic pressure and centerline area fraction that resulted in these fits are shown in Fig. 5. Our analysis shows that acoustic pressure is almost linearly proportional to the input voltage (Fig. 5A), which is expected based on scaling models of acoustophoretic transport.^39,40^ The centerline area fraction increases at a rate of ∼0.86% for every volt applied to the acoustofluidic device (Fig. 5B). While we prepare all of our samples with the same procedures to minimize experimental variability, the introduction of particles to the device is stochastic. This could explain the variation we observe in centerline area fraction. The variability in particle concentration does not appear to influence the acoustic pressure as significantly, with only some deviation from linear behavior observed for 60 V, 70 V and 80 V. The coefficient of determination (*r*^2^) for the regression lines shown in Fig. 5 are 0.97 for the acoustic pressure and 0.75 for the centerline area fraction. Overall, this suggests that, despite the variability in centerline concentration, our model does predict the expected linear relationship between voltage and acoustic pressure. This approach also provides an alternative way to determine acoustic pressure by examining the particle density distribution about the acoustic node.

**Fig. 6:**
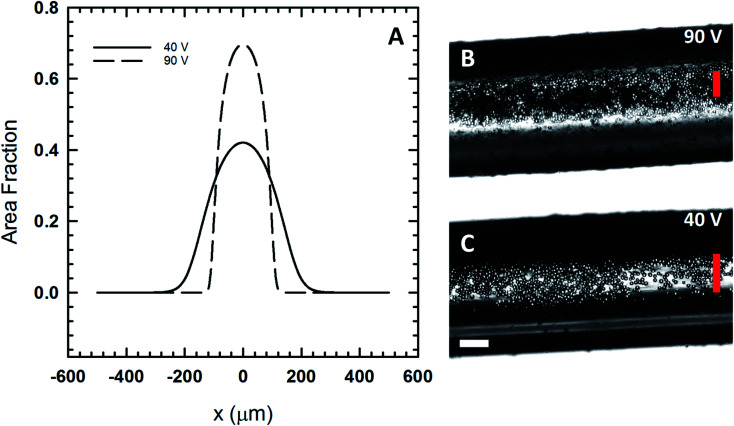
(A) The expected area fraction distribution in the print head using eqn (8) and the viscosity of the polymeric fluid. (B) A polymer Janus fiber assembled at 90 V show the compression of the particles in the center of the fiber. (C) A fiber assembled at a lower voltage, 40 V, shows the particles are more widely distributed. The vertical red lines show the predicted full-width-at-half-maximum for both cases based on the model. The horizontal scale bar is 200 μm.

With the acoustic pressure measured in our device, we apply the suspension balance model to the polymeric fluid consisting of the UV resin, acetone and isopropyl alcohol mixed in a 1 : 1 : 1 mass ratio by modifying the fluid viscosity (1.77 mPa s) and particle radius (29.5 μm diameter for PMMA filler particles). These parameters have the effect of decreasing acoustic Peclet number. The experimentally measured centerline concentration values were used to estimate the centerline concentration for the polymer case. Using the values for Peclet number and centerline concentration for the model, we evaluated the density distribution for two different voltage inputs (Fig. 6A) where the system consists of PMMA filler particles dispersed in the polymeric fluid. The full-width-at-half-maximum (FWHM) is the point at which the local area fraction drops to half the centerline area fraction. Based on our calculations in Fig. 6A, we predict that the FWHM for the 90 V case is 176 μm, while the FWHM for the 40 V case is 266 μm. The experimental images (Fig. 6B and C) show filler particles assembled and immobilized in a UV cured fiber that was extruded from our 3D printer under the voltage conditions described here.

The 90 V case (Fig. 6B) shows that the particles are largely confined to the region defined by the FWHM (vertical red line). In this case, we see that the acoustic pressure is sufficiently high so as to cause particles to form a multilayer. While the model is not able to capture the multilayer formation, it does closely predict the width of the region to which the particles are confined to. At 40 V, the model predicts far less packing and a microstructure that is more distributed out based on the FWHM. Experimental results at 40 V (Fig. 6C) exhibit far less packing in comparison to the 90 V case. In this case, the particles are more widely distributed with assembly occurring near the fiber edge where light scattering makes it difficult to image them directly.

**Fig. 7:**
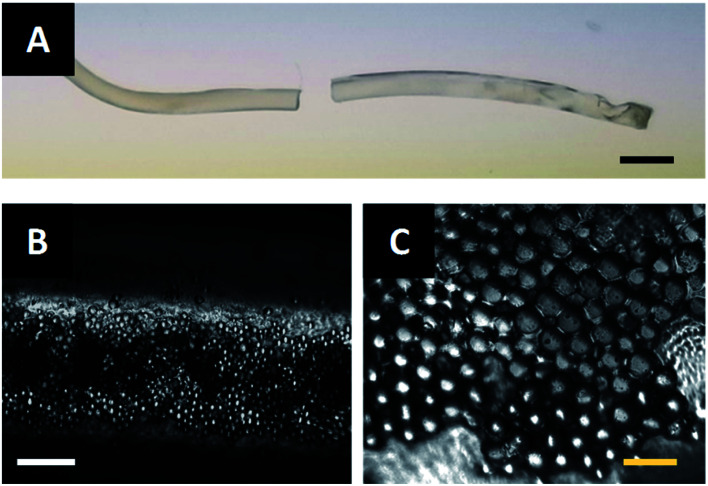
(A) A Janus fiber extruded from the acoustofluidic device. Scale bar is 2 mm. (B) A 10× optical micrograph (100 μm scale bar) of the internally assembled PMMA particles and (C) a 40× magnification (30 μm scale bar) of the assembled PMMA particles.

The fibers described in Fig. 6B and C are extruded through the acoustic print head under the conditions described in Materials and methods. After assembling at the acoustic node, the particle-polymer dispersion passes through the acoustic print head and is deposited to a microscope slide place inside the UV illumination chamber. The extruded fiber (Fig. 7A) has a mean diameter of 0.94 mm and cures completely in approximately two-and-a-half minutes. Observation under a microscope shows that the PMMA particles are internally assembled at the center of Janus fiber (Fig. 7B). These particles form a layer at the bottom of the fiber due to sedimentation, which is consistent with observations from our previous publication.^27^Fig. 7C shows a 40× magnified image of the assembled filler particle microstructure.

**Fig. 8:**
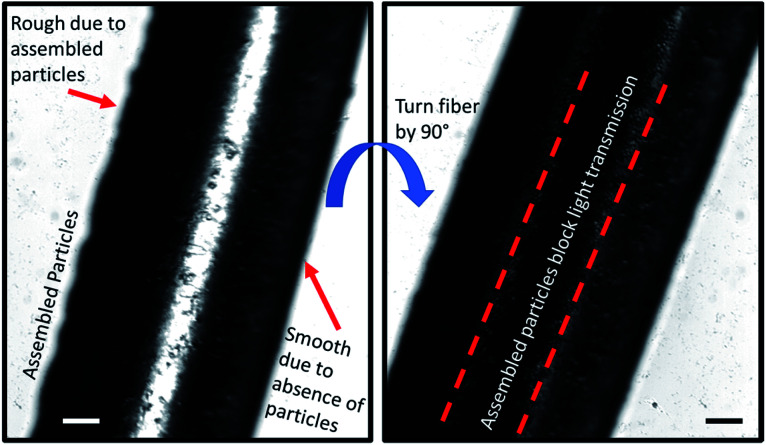
Sample images taken from a supplemental video showing the structure of the Janus fiber observed under an optical microscope. (Left) The Janus fiber turned on its side exhibits roughness where particles are assembled, while the side without particles is smooth. (Right) When the Janus fiber is rotated 90°, the transmission of light through the fiber decreases due to the presence of assembled particles. Scale bars are 200 μm.

In order to image the structure of the Janus fiber, a sample was placed on an Olympus IX70 microscope stage with images captured with a sCMOS camera. The Janus fiber was rotated on the microscope stage to show that the side without assembled particles exhibits a smooth structure (Fig. 8). The side of the Janus fiber containing particles exhibits greater degree of roughness, which is consistent with particles that have settled to the bottom of the fiber during the assembly process. When the fiber is rotated 90°, the particles are assembled in sufficient concentration to significantly block the transmission of light through the Janus fiber. A video of the rotation process is available as a ESI.†

**Fig. 9:**
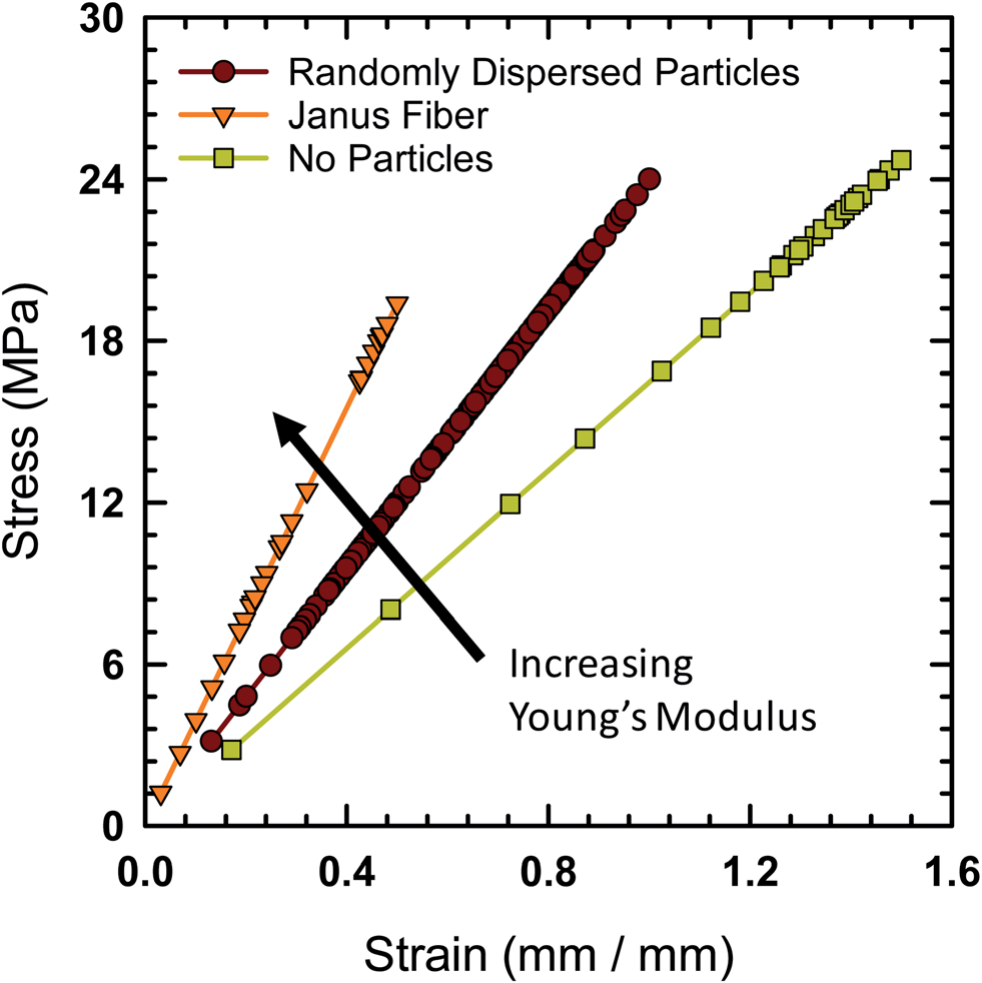
Engineering stress as a function of strain for three fiber types (polymer fiber with no particles, polymer fiber with randomly dispersed particles and the Janus fiber). The slope of the data increases from right to left, indicating that the Janus fiber features the largest Young's modulus.

Tensile testing was performed on the Janus fibers extruded from the acoustic print head in order to characterize their mechanical properties. A Mark-10 test stand (Model ES-20) with a 50 lbf digital force gauge (Mark-10, Model M5-50) was used to perform tensile measurement. The fibers were cut to an initial length of 12.7 mm prior to placement on the test stand. The Young's modulus of the fibers is calculated using standardized protocol TM 2.4.18.2 recommended by the IPC association.^41^Fig. 9 shows the engineering stress experienced by the Janus fiber as a function of strain. The Young's modulus of the different fibers tested with this protocol is equal to the slope extracted from these curves.

The fiber, in the absence of filler particles (*i.e.*, bare polymer), is calculated to have a Young's modulus of 24.7 MPa based on data from Fig. 9. The addition of a random distribution of particles in the absence of an acoustic field creates a polymer-particle composite with a Young's modulus of 29.6 MPa. The Young's modulus as a function of concentration for a dilute dispersion of filler particle in the polymer matrix is,^42^*E*/*E*_o_ = 1 + 2.5*ϕ*_v_, where *E* is the Young's modulus of the composite, *E*_o_ is the Young's modulus of the polymer without particles and *ϕ*_v_ is the volume fraction. Using the measured Young's modulus for the polymer fiber without particles and a volume fraction of 7.8%, we find that the calculated value of *E* = 29.5 MPa, which closely agrees with the measured value for the composite fiber when it contains randomly distributed particles.

The Young's modulus for the Janus fiber with assembled particles is measured to be 50.5 MPa, which is a factor of two larger than the polymer fiber in the absence of particles. Despite using the same volume fraction as the randomly distributed sample, this increase in Young's modulus appears to occur as a result of using the acoustic field to concentrate the particles into a close-packed configuration. This result suggests that filler particle microstructure (*i.e.*, particle distribution) is critical for determining the mechanical properties of the resulting composite.

Drawing upon the analogous relationship between Hookean elasticity and Newtonian viscosity,^43^ we examine a model for Young's modulus that incorporates the effect of filler particle microstructure. The model, based on an analysis of Newtonian viscosity for a filler particle dispersion at zero shear, is approximately ,^44^9
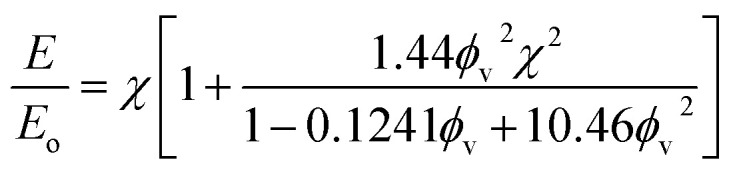
where *χ* is the value of the radial distribution function (RDF) for the filler particles at contact (*i.e.*, the first peak in RDF). Since the Young's moduli and bulk volume fraction are known, we can use eqn (9) to find the values of *χ* for both fiber with randomly distributed particles and the Janus fiber. This process allows us to find that *χ* is 1.18 for the randomly distributed particles and 1.98 for the Janus fiber. In our ESI,† we show that the *χ* value can be derived by using the Carnahan–Starling equation of state for hard spheres, where,10
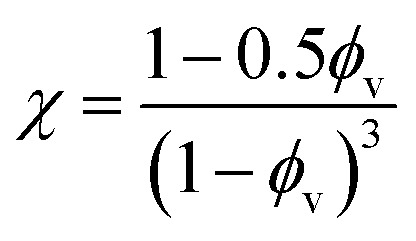


Evaluating *χ* with eqn (10) using the bulk volume fraction yields a value of 1.23, which closely agrees with the value found for the randomly distributed sample. The lower value for *χ* in the randomly distributed sample suggests that the interactions between particles in the composite are slightly softer than would be expected for hard spheres. The larger value of *χ* for the acoustically assembled sample is consistent with an increase in filler particle contact due to acoustic concentration.

## 5. Conclusion

In this paper, we demonstrate that acoustic assembly-based additive manufacturing can be used to fabricate millimeter-scale Janus fibers. We developed a suspension balance model to demonstrate how the acoustic print head on our 3D printing system operates. Acoustic assembly experiments were performed in water to calibrate the model. These measurements exhibited a linear relationship between acoustic pressure and voltage, which is expected based on acoustophoretic models. This approach could be used in other acoustophoretic experiments to estimate pressure based particle density distribution about the acoustic node. The model showed good agreement with observed particle distribution when applied to acoustic assembly in a UV curable polymer resin. Acoustophoretic transport, aided by sedimentation, forms a structured Janus fiber with PMMA particles embedded on one side of the fiber after curing. Rotating the fiber on a microscope stage shows that the side containing particles is rough. Further rotation of the Janus fiber exhibits a reduction in light transmission, a quality that could potentially be useful for optical encoding and identification or serve as attachment sites for chemical sensors. Tensile testing of the Janus fiber measured a Young's modulus of 50.5 MPa, which is approximately twice as high as the 24.7 MPa measured for the polymer fiber without particles. The inclusion of randomly dispersed particles only increases the Young's modulus of the fiber to a value of 29.6 MPa, which indicates that the process of concentrating particles with an acoustic field is responsible for the difference in Young's modulus. Based on the analogous relationship between Hookean elasticity and Newtonian viscosity, we show that the nearest neighbor particle distribution likely plays a role in determining the mechanical properties of the Janus fiber. The process described here could serve as a multimaterial additive manufacturing platform for the fabrication of a variety of functional Janus fibers with possible applications to wearable textiles, soft robotics or surgical sutures. In future work, we aim to perform a systematic study of filler particle microstructure to determine the fundamental connection between this parameter and bulk mechanical properties.

## Conflicts of interest

There are no conflicts to declare.

## Supplementary Material

RA-010-C9RA09796A-s001

RA-010-C9RA09796A-s002
