# Supplementary material for: Acoustophoretic assembly of millimeter-scale Janus fibers
Source: RSC Adv. 2020 Jan 2;10(1):434–43. doi: 10.1039/c9ra09796a (PMC9047537; doi:10.1039/c9ra09796a)
Supplement: RA-010-C9RA09796A-s002 [file RA-010-C9RA09796A-s002.pdf]

Electronic Supplementary Material (ESI) for RSC Advances.
This journal is © The Royal Society of Chemistry 2019

Supplementary Document

Particle Type	Size Range (microns)	Vendor	Product Number
Poly(Methyl Methacrylate)	27-32	Cospheric	PMPMS-1.2 27-32um
Copper	20	US Research Nanomaterials	US5002
Stainless Steel 316L	20	US Research Nanomaterials	US316L3D
SrAl2O3	50	Techno Glow	P02-GRN-M004Z
Graphite Powder	20	US Research Nanomaterials	US1158M

Table 1 – A list of particle types tried as filler particles and their associated product information.

3D Printing of $SrAl_2O_3$: Eu,Dy - UV Resin composite

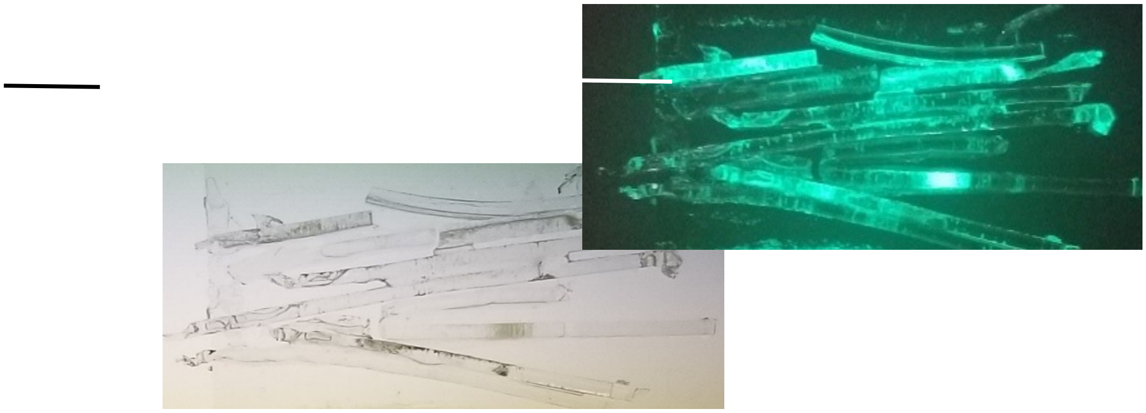

Figure S1. A) the 3D printed self-assembled Strontium Aluminate- UV resin fibers in a raster scan and B) the luminescence of the sample in the dark. Scale bar is 1cm.

The $SrAl_2O_3$:Eu, Dy microparticles are suspended in the UV resin solution assemble at a flow rate of approximately 2-5 ml/hr due to handheld input, an applied voltage of 95 V_{pp} and a frequency of 830 KHz. The UV resin (10:1:1 UV Resin: Isopropyl Alcohol: Acetone) is cured in

UV light for 4 minutes and 30 seconds to freeze the crystals as shown in figure S1A. A step size of 1mm is used in the X and Y direction and the Z-direction (height of the nozzle) is kept constant. The X-Y motion is controlled using Arduino motors using Universal Gcode Sender interface. The G-code was manually entered to obtain a raster scan. The cell is placed such that only the outlet end is exposed to the UV light.

We extracted a sample polymer fiber that is 5cm in length and 0.94 mm in diameter as shown in Figure 3A and the luminescent 3D printed self-assembled particle-polymer layer can be seen in glowing in the dark in Figure S1B. Since Strontium Aluminate is luminescent, the particle arrangement in the threads can be seen by the green hue in the dark. Strontium Aluminate loses its luminescence after 24 hours and can be recharged using solar power, demonstrating the ability of this technique to fabricate new materials with varying microstructures using rapid prototyping.

The $SrAl_2O_3$:Eu, Dy microparticles are suspended in the UV resin solution assemble at a flow rate of approximately 2-5 ml/hr due to handheld input, an applied voltage of 95 V_{pp} and a frequency of 830 KHz. The UV resin (10:1:1 UV Resin: Isopropyl Alcohol: Acetone) is cured in UV light for 4 minutes and 30 seconds to freeze the crystals as shown. We extracted a sample polymer fiber that is 5cm in length and 0.94 mm in diameter. The 4X image of the fiber shows continuous close-packed structures can be observed to form a single layer around the polymer fiber as shown in Figure 3B. Since Strontium Aluminate is luminescent, the particle arrangement in the threads can be seen by the green hue in the dark.

The graphite microflakes (plates) are suspended in the UV resin solution assemble at a flow rate of 2 ml/hr due to handheld input, an applied voltage of 95 V_{pp} and a frequency of 830 KHz. The UV resin is cured in UV light for 3 minutes to freeze the crystals as shown. We extracted a sample polymer fiber that is 8cm in length and 0.94 mm in diameter. The 4X image of the fiber

shows continuous, close packed network (structure) of graphite can be observed to form a single layer of graphite at the bottom of the polymer fiber. The graphite network can also be observed with the naked eye as demonstrated in Figure S2.

Copper microparticles (dry powder) are suspended in the UV resin solution assemble at a flow rate of approximately 2-5 ml/hr due to handheld input, an applied voltage of 95 V_{pp} and a frequency of 830 KHz. The UV resin (10:1:1 UV Resin: Isopropyl Alcohol: Acetone) is cured in UV light for 4 minutes and 30 seconds to freeze the crystals as shown. We extracted a sample polymer fiber that is 7cm in length and 0.94 mm in diameter. The 1X image shown in Figure S2 of the fiber shows continuous Copper deposition as a thin line can be observed on the polymer fiber.

Stainless steel microparticles are suspended in the UV resin solution assemble at a flow rate of 2 ml/hr due to handheld input, an applied voltage of 95 V_{pp} and a frequency of 830 KHz. The UV resin (10:1:1 UV Resin: Isopropyl Alcohol: Acetone) is cured in UV light for 4 minutes and 30 seconds to freeze the crystals as shown in Figure S2. We extracted a sample polymer fiber that is 5cm in length and 0.94 mm in diameter. The 1X image of the fiber shows close-packed structures can be observed to form a single layer around the polymer fiber.

The particle assembly demonstrated using PMMA beads is not as uniform as seen in the polystyrene bead assembly as the dry, unfunctionalized PMMA particles are suspended in the resin solution and so, they do not form a stable solution as opposed to the functionalized polystyrene solution we used. The PMMA beads are not monodisperse and the difference in particle sizes causes a non-hexagonal packing of the packing. Similar is the case for the non-uniform structure formed using the non-uniform shape of $SrAl_{2O}3$:Eu, Dy particles and graphene flakes. Also, due to the high weight of $SrAl_2O_3$:Eu, Dy and Copper, the particles would settle down in the syringe.

So these samples were handheld vertically to make sure that the concentration of the colloidal solution did not vary drastically over time.

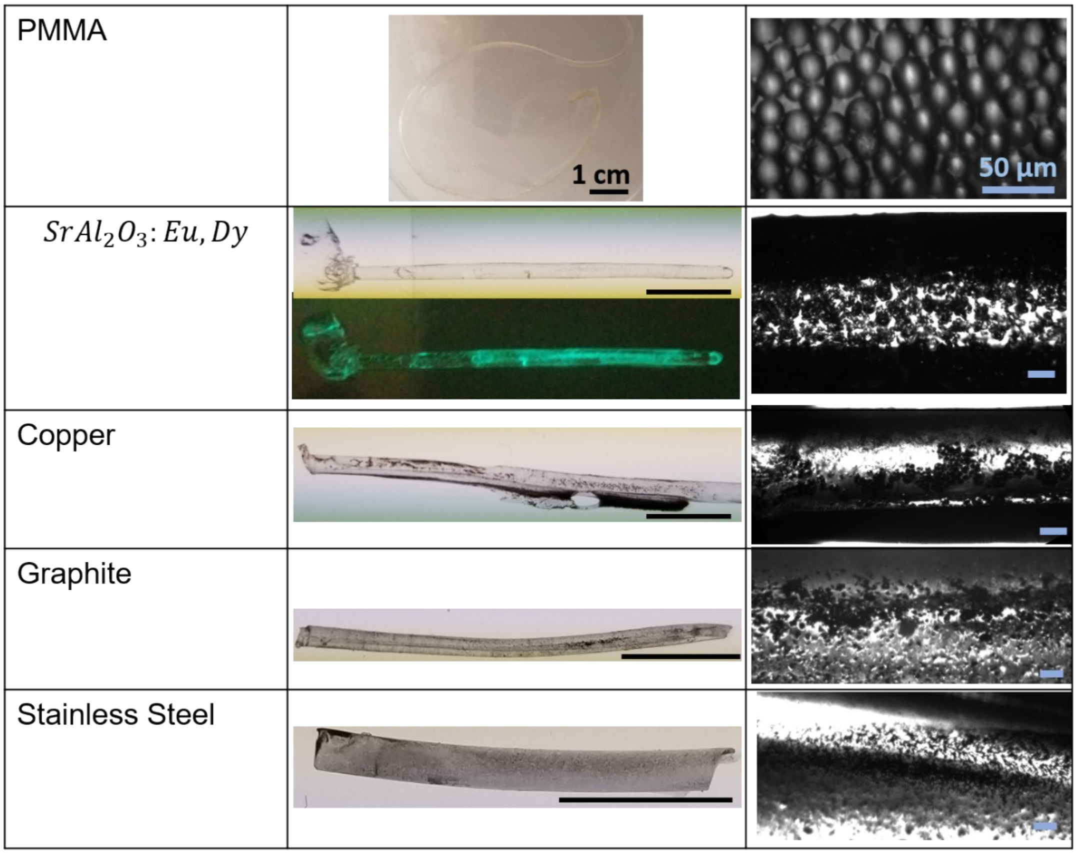

Figure S2. Self-assembled Microparticle-UV resin fibers and the corresponding microstructures for PMMA, $SrAl_2O_3$: Eu, Dy; Copper, graphite and stainless steel. The microstructures of the corresponding fibers is shown. Black scale bars are 1 cm and blue scale bars are 50 µm.

Derivation of Radial Distribution Function at Contact

The relationship between energy interaction strength, particle size and osmotic pressure is[1],

$$\Pi = \frac{\phi_v}{V_p} k_b T - \frac{2\pi}{3}\left(\frac{\phi_v}{V_p}\right)^2 \int_0^\infty r^3 g(r) \frac{dU}{dr} dr$$

where g(r) is the non-dimensional radial distribution as a function of position and U is the energy associated with the interparticle interactions. If we assume that only hard sphere interactions are important for particles embedded in the polymer matrix, then dU/dr is a delta function such that[2],

$$\frac{dU}{dr} = -k_b T\,\delta(r-2a)$$
$$\delta(r-2a) = \begin{cases} 0 & r > 2a \\ 1 & r = 2a \end{cases}$$

Applying this relationship to the osmotic pressure term we have,

$$\Pi = \frac{\phi_v}{V_p} k_b T + \frac{2\pi}{3}\left(\frac{\phi_v}{V_p}\right)^2 k_b T \int_0^\infty r^3 g(r)\,\delta(r-2a)\,dr$$

Properties of the delta function[3] allow us to express this equation as,

$$\Pi = \frac{\phi_v}{V_p} k_b T + \frac{2\pi}{3}\left(\frac{\phi_v}{V_p}\right)^2 k_b T\,(2a)^3 g(2a)$$

By diving through $\phi_v k_b T / V_p$ and using the volume of a sphere, this expression reduces to,

$$Z = \frac{\Pi V_p}{\phi_v k_b T}$$
$$Z = 1 + 4\phi_v \chi$$

where $\chi = g(2a)$ is defined in our manuscript as the radial distribution at contact. This derivation shows us that χ for a randomly distributed sample of hard spheres only depends on the equation of state selected for Z and the bulk volume fraction. If we select the Carnahan-Starling equation of state[4], $Z = \left(1 + \phi_v + \phi_v^2 - \phi_v^3\right)\left(1 - \phi_v\right)^{-3}$, and evaluate Z using the bulk volume

fraction, then χ should only depend volume fraction. Substituting the Carnahan-Starling equation of state into the expression above yields Eq. 10 in our manuscript.

$$\chi = \frac{1 - 0.5\phi_v}{(1 - \phi_v)^3}$$

This expression is valid for a randomly distributed sample of hard spheres. Our measurements in samples with randomly distributed particles show that our experimental estimate for χ based on mechanical measurements is close to the value of χ calculated using Eq. 10.

References

1 J. F. Brady, *Journal of Chemical Physics*, 1993, **98**, 3335–3341.
2 D. M. Heyes, *The Liquid State: Applications of Molecular Simulations*, Wiley, Chichester ; New York, 1 edition., 1998.
3 E. W. Weisstein, Delta Function, http://mathworld.wolfram.com/DeltaFunction.html, (accessed December 16, 2019).
4 N. F. Carnahan and K. E. Starling, *Journal of Chemical Physics*, 1969, **51**, 635-.
